# The chaperone-assisted selective autophagy complex dynamics and dysfunctions

**DOI:** 10.1080/15548627.2022.2160564

**Published:** 2023-01-03

**Authors:** Barbara Tedesco, Leen Vendredy, Vincent Timmerman, Angelo Poletti

**Affiliations:** a Laboratory of Experimental Biology, Dipartimento di Scienze Farmacologiche e Biomolecolari, Dipartimento di Eccellenza 2018-2027, Università degli studi di Milano, Milan, Italy; b Unit of Medical Genetics and Neurogenetics, Fondazione IRCCS Istituto Neurologico Carlo Besta, Milan, Italy; c Peripheral Neuropathy Research Group, Department of Biomedical Sciences, Institute Born Bunge, University of Antwerp, Antwerpen, Belgium

**Keywords:** Aggresome, BAG3, HSPA, HSPB8, misfolding, myopathy, neurodegenerative diseases, neuropathy, proteostasis, SQSTM1, STUB1

## Abstract

Each protein must be synthesized with the correct amino acid sequence, folded into its native structure, and transported to a relevant subcellular location and protein complex. If any of these steps fail, the cell has the capacity to break down aberrant proteins to maintain protein homeostasis (also called proteostasis). All cells possess a set of well-characterized protein quality control systems to minimize protein misfolding and the damage it might cause. Autophagy, a conserved pathway for the degradation of long-lived proteins, aggregates, and damaged organelles, was initially characterized as a bulk degradation pathway. However, it is now clear that autophagy also contributes to intracellular homeostasis by selectively degrading cargo material. One of the pathways involved in the selective removal of damaged and misfolded proteins is chaperone-assisted selective autophagy (CASA). The CASA complex is composed of three main proteins (HSPA, HSPB8 and BAG3), essential to maintain protein homeostasis in muscle and neuronal cells. A failure in the CASA complex, caused by mutations in the respective coding genes, can lead to (cardio)myopathies and neurodegenerative diseases. Here, we summarize our current understanding of the CASA complex and its dynamics. We also briefly discuss how CASA complex proteins are involved in disease and may represent an interesting therapeutic target.

**Abbreviation** ALP: autophagy lysosomal pathway; ALS: amyotrophic lateral sclerosis; AMOTL1: angiomotin like 1; ARP2/3: actin related protein 2/3; BAG: BAG cochaperone; BAG3: BAG cochaperone 3; CASA: chaperone-assisted selective autophagy; CMA: chaperone-mediated autophagy; DNAJ/HSP40: DnaJ heat shock protein family (Hsp40); DRiPs: defective ribosomal products; EIF2A/eIF2α: eukaryotic translation initiation factor 2A; EIF2AK1/HRI: eukaryotic translation initiation factor 2 alpha kinase 1; GABARAP: GABA type A receptor-associated protein; HDAC6: histone deacetylase 6; HSP: heat shock protein; HSPA/HSP70: heat shock protein family A (Hsp70); HSP90: heat shock protein 90; HSPB8: heat shock protein family B (small) member 8; IPV: isoleucine-proline-valine; ISR: integrated stress response; KEAP1: kelch like ECH associated protein 1; LAMP2A: lysosomal associated membrane protein 2A; LATS1: large tumor suppressor kinase 1; LIR: LC3-interacting region; MAP1LC3/LC3: microtubule associated protein 1 light chain 3; MTOC: microtubule organizing center; MTOR: mechanistic target of rapamycin kinase; NFKB/NF-κB: nuclear factor kappa B; NFE2L2: NFE2 like bZIP transcription factor 2; PLCG/PLCγ: phospholipase C gamma; polyQ: polyglutamine; PQC: protein quality control; PxxP: proline-rich; RAN translation: repeat-associated non-AUG translation; SG: stress granule; SOD1: superoxide dismutase 1; SQSTM1/p62: sequestosome 1; STUB1/CHIP: STIP1 homology and U-box containing protein 1; STK: serine/threonine kinase; SYNPO: synaptopodin; TBP: TATA-box binding protein; TARDBP/TDP-43: TAR DNA binding protein; TFEB: transcription factor EB; TPR: tetratricopeptide repeats; TSC1: TSC complex subunit 1; UBA: ubiquitin associated; UPS: ubiquitin-proteasome system; WW: tryptophan-tryptophan; WWTR1: WW domain containing transcription regulator 1; YAP1: Yes1 associated transcriptional regulator

## Introduction

Autophagy is a cytoplasmic route for the degradation of long-lived proteins, large multimeric assemblies, aggregates, and dysfunctional damaged organelles. Autophagy divides into three main branches: i) macroautophagy (referred to as autophagy herein), ii) microautophagy and iii) chaperone-mediated autophagy (CMA), which differ in the mechanisms through which substrates are internalized into lysosomes [1–3]. Autophagy has long been considered a nonspecific “in-bulk” process for recycling cellular components in response to starvation, thereby compensating for the lack of nutrients [4]. However, it has become clear that autophagy also contributes to maintaining intracellular homeostasis in the absence of starvation (or other stress), by selectively degrading cargo material like aggregated proteins, damaged organelles or invading pathogens [5–7]. Selective autophagy pathways involve proteins and specialized autophagic receptors which recognize the cargo, often mediated by cargo ubiquitination [8,9]. When bound to the cargoes, autophagy receptors target and bridge them to the phagophore-associated members of the MAP1LC3/LC3 (microtubule associated protein 1 light chain 3) or GABARAP (GABA type A receptor-associated protein) subfamilies for autophagic cargo engulfment [10–14].

**Figure 1.**
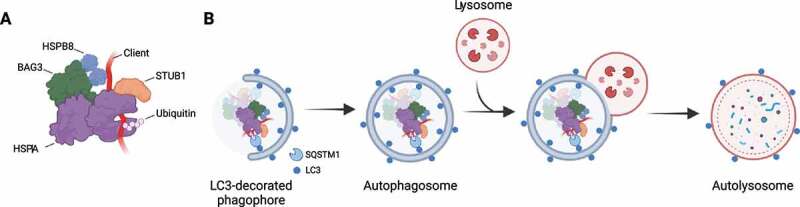
The chaperone-assisted selective autophagy (CASA) complex. (A) Schematic representation of the CASA complex. BAG3 (green) mediates the assembly of the CASA complex, thereby linking chaperones of the HSPA family (purple) to small heat shock proteins such as HSPB8 (blue). The HSPA-associated E3 ubiquitin ligase STUB1 (orange) interacts with the complex for the ubiquitination of the bound client protein. (B) Engulfment of the CASA complex by LC3-decorated phagophore membrane is facilitated by the autophagic cargo receptor SQSTM1, leading to formation of double-membrane vesicles (called autophagosomes) and final degradation of its content by fusion with the lysosomes.

A selective autophagic route utilized to remove damaged and misfolded proteins is chaperone-assisted selective autophagy (CASA). CASA relies on the formation of a heteromeric complex, originally described in muscle cells, consisting of the heat shock proteins (HSPs) HSPA/HSP70 (heat shock protein family A (Hsp70)) and HSPB8 (heat shock protein family B (small) member 8), the cochaperone BAG3 (BAG cochaperone 3), and the E3 ubiquitin ligase STUB1/CHIP (STIP1 homology and U-box containing protein 1), although the latter might be replaced by other E3 ubiquitin ligases (Figure 1) [15]. Although all these proteins are widely expressed across tissues, higher expression levels of some members of the CASA complex are described in striated muscle cells and neurons, where CASA plays a pivotal role in cellular homeostasis [16–18]. Indeed, a dysfunctional CASA complex causes a broad range of diseases including (cardio)myopathies, neuropathies and neurodegenerative diseases. Here, we aim to describe the current knowledge on the dynamics of the CASA complex and its role in autophagy and beyond. Then, the CASA complex activities in physiology and pathology will be briefly summarized.

## CASA complex members

The term “CASA” was originally introduced by Arndt et al. [15] to define a protein degradation pathway mediated by BAG3. In this pathway, the role of BAG3, as a scaffold, is to physically link the HSPAs to HSPBs, thus promoting client refolding or degradation. Although it cannot be excluded that other HSPBs may take part in the CASA complex, HSPB8 displays the highest binding affinity for BAG3 [19]. In this view, CASA-mediated degradation primarily refers to those processes that rely on the BAG3-HSPA-HSPB8 ternary complex for the autophagy-lysosomal clearance of a certain substrate. The CASA complex also involves other factors for substrate handling, such as ubiquitin ligases. Among the HSPA-associated ubiquitin ligases, STUB1 was identified in the CASA complex, although its function may be accomplished by other ubiquitinating enzymes.

### HSPAs

**Figure 2.**
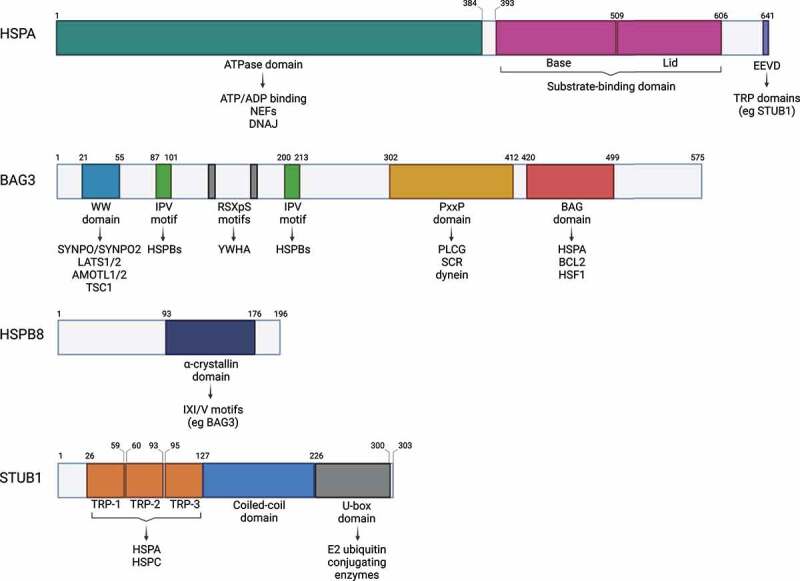
CASA members with their protein domains and corresponding interactors. Schematic representation of the protein domains of HSPA, BAG3, HSPB8 and STUB1 and related interactors.

**Figure 3.**
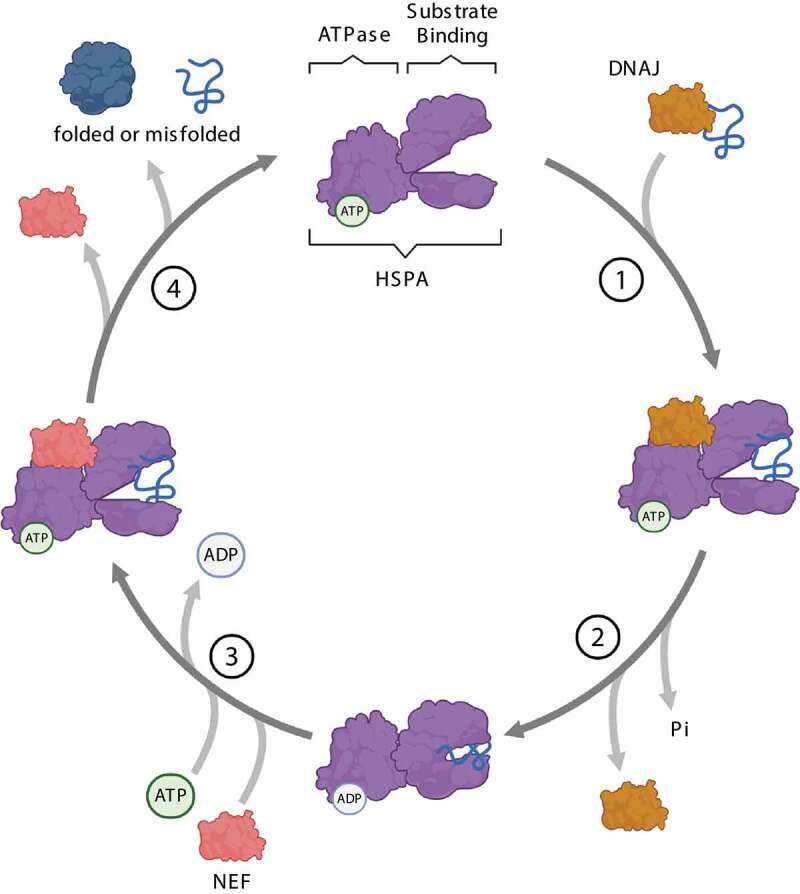
Steps of the HSPA substrate binding/release cycle. (1) DNAJ (orange) mediates the delivery of nascent or misfolded proteins to ATP-bound HSPA (purple). (2) Hydrolysis of ATP to ADP results in a conformational change of HSPA: the “lid” of the substrate-binding domain closes to tightly bind the substrate. DNAJ dissociates from the chaperone complex. (3) A nucleotide exchange factor (NEF, pink) binds to HSPA, facilitating the exchange of ADP for ATP. (4) ATP binding to the ATPase domain induces the opening of the “lid”, thereby enabling substrate release from HSPA.

HSPA is a family of ~70-kDa chaperones serving as central hubs of the protein quality control (PQC) system [20]. HSPAs bind a wide array of proteins and promote their folding, refolding, delivery to organelles or degradation. The HSPA family members are defined as “foldases”, meaning they exert ATPase activity to promote substrate folding. Structurally, HSPAs are composed of two domains: an N-terminal ATPase domain for ATP binding, and a C-terminal domain for substrate interaction (Figure 2). The substrate-binding domain, formed by two subdomains called the base and the lid, can assume an open or a closed conformation. The substrate-binding and ATPase domains are characterized by an allosteric interaction: ATP binding induces the open conformation of the substrate-binding domain by separating the base and the lid, thus allowing the binding of the client protein. In turn, client protein binding to the open conformation of the substrate-binding domain induces a conformational change at the ATPase domain interface and ATP hydrolysis. By getting closer, the base and the lid entrap the client protein with high affinity and promote its folding [21,22] (Figure 3). The activity of HSPAs is modulated by other chaperones, such as DNAJ/HSP40/J-domain proteins/JDPs, and co-chaperones, such as nucleotide exchange factors. DNAJs target the client proteins to HSPA and potentiate the closed conformation by stimulating the ATPase activity and efficient locking of the substrate [23]. Instead, nucleotide exchange factors, such as the co-chaperones of the BAG (BAG cochaperone) family, favor the closed-to-open conformational change, by stimulating ADP dissociation from the ATPase domain for client protein release.

HSPAs represent a platform for client proteins and interacting partners to determine the fate toward folding or disposal. For instance, by cooperating with HSP90 (heat shock protein 90), HSPAs promote the folding of neo-synthesized proteins while, by cooperating with HSPBs/small heat shock proteins and HSPH1/HSP100 (heat shock protein family H (Hsp110) member 1), HSPAs favor the disaggregation of protein aggregates [24,25]. In addition, based on interacting co-chaperones and clients, HSPAs cooperate with multiple degradative pathways: i) the ubiquitin-proteasome system (UPS), by forming a protein complex with STUB1 and BAG1, or by interacting with BAG2 for the ubiquitin-independent delivery of substrates to the proteasome [26,27]; ii) the CASA pathway, by interacting with STUB1, BAG3 and HSPB8 [15]; iii) CMA, by binding substrates carrying a KFERQ-like motif and the LAMP2A (lysosomal associated membrane protein 2A) protein complex located in the lysosomal membrane [28–30] and iv) selective microautophagy by the ESCRT machinery [30,31]. Notably, the HSPA family comprises 13 members, which differ in expression (inducible/constitutive or ubiquitous/cell-specific), intracellular localization (cytoplasmic/associated with organelles) and function [32,33]. Among HSPA members, the constitutive HSPA8/HSC70, and the inducible HSPA1A, have been mainly investigated in the cytosolic PQC and the CASA complex [15,34].

### BAG3

BAG3 is an ~75-kDa co-chaperone ubiquitously expressed, but to higher levels in cardiac and skeletal muscles as well as in cancer cells [16,35–38]. BAG3 belongs to the BAG family, which includes six members (BAG1 to BAG6). The first identified member of the BAG family, BAG1, was initially described as an interactor of the anti-apoptotic protein Bcl-2 [37,39,40]. The other BAG family members were identified through a yeast two-hybrid screen assay as interactors of the ATPase domain of the HSPA chaperone. Indeed, all BAG members contain at least one copy of a BAG domain, which comprises 78–120 amino acids and is responsible for the interaction with HSPA [41–43]. Among BAGs, BAG3/Bis/CAIR-1 has the highest affinity for cytoplasmic HSPA [44]. Oxidative stress, proteasome inhibition, heat shock and other stressors enhance BAG3 expression [45–48], mainly resulting from the activation of HSF1 (heat shock transcription factor 1) [46,47,49,50]. In turn, BAG3 was recently shown to interact with HSF1 via its BAG domain, thereby regulating the nucleocytoplasmic shuttling of HSF1 upon heat stress [51]. In cells, BAG3 is mainly cytosolic but relocates to the perinuclear region after acute stress [52,53].

Besides the BAG domain, BAG3 possesses additional domains which mediate its binding to multiple partners involved in various intracellular pathways: a tryptophan-tryptophan (WW) domain, a proline-rich (PxxP) region, two RSXpS motifs and two isoleucine-proline-valine (IPV) motifs [19,43,54,55] (Figure 2). The WWdomain at the N-terminus is responsible for interactions with proteins containing proline-rich regions. These include the synaptopodin proteins SYNPO and SYNPO2; LATS1 (large tumor suppressor kinase 1)-LATS2 and AMOTL1 (angiomotin like 1)-AMOTL2; TSC1 (TSC complex subunit 1), an inhibitor of MTOR (mechanistic target of rapamycin kinase) [56–60]. The two IPV motifs of BAG3 are responsible for the interaction with HSPBs, primarily HSPB8 [19]. Through its PxxP domain, BAG3 recognizes SH3-domain containing proteins, such as PLCG/PLCγ (phospholipase C gamma), SRC, and the dynein motor complex [52,54,61]. The RSXpS (RSQS136 and RSQS173) motifs are recognized by the YWHA/14–3-3 proteins, which couple BAG3 to the dynein-intermediate chain of the dynein machinery [62]. Recently, putative LC3-interacting regions (LIRs) have been described throughout BAG3 based on the analysis of its protein sequence, suggesting that BAG3 itself might be able to directly interact with autophagosome membranes [63].

### HSPB8

HSPB8 is an ~22-kDa chaperone ubiquitously expressed, especially in cardiac and skeletal muscles. HSPB8 belongs to the mammalian HSPB family which comprises 10 members (HSPB1-HSPB3, CRYAA/HSPB4, CRYAB/HSPB5, HSPB6-HSPB9, and ODF1/HSPB10) all sharing a conserved 80–90 amino acids long α-crystallin domain, while the N- and the C-terminal domains are more variable (Figure 2) [64,65]. HSPBs are ATP-independent holdases which interact with misfolded substrates, preventing aggregation [66]. For the ATP-driven refolding process, HSPBs indirectly interact with ATP-dependent chaperones with foldase activity, such as HSPAs [67,68]. While some HSPBs display dynamic hetero- and homo- oligomerization, HSPB8 mainly forms homodimers and weakly interacts with other HSPBs. Through a pocket formed by the β4 and β8 sheets of its α-crystallin domain [19], HSPB8 binds to the IPV motif of the co-chaperone BAG3, with which it forms a 2:1 stoichiometric complex [19,69], stabilizing HSPB8 under physiological, baseline conditions [70,71]. Similar to BAG3, expression of HSPB8 is induced by stress, mainly proteotoxic stress, but also oxidative and osmotic stress [72–74].

### STUB1

STUB1 is an ~34.5-kDa E3 ubiquitin ligase highly conserved across species [75]. STUB1 possesses three pairs of tetratricopeptide repeats (TPRs) at the N-terminus and a 90-amino acids long catalytic U-box domain at the C-terminus. In its central region, STUB1 presents a coiled-coil domain involved in its dimerization and stability (Figure 2) [75,76]. Through its TPRs, STUB1 interacts with HSPA and HSPC, and through the U-box domain, it binds E2 ubiquitin-conjugating enzymes [75–77], allowing to be at the crossroad for chaperone-bound substrate ubiquitination and routing clients either to proteasomal or to autophagic degradation [78]. Indeed, ubiquitin chains generated by STUB1 may involve different types of lysine (K)-linkage, which include the K6, K27, K48, K63 [79–81]. Ubiquitination at K48 or K63 targets substrates to the UPS or autophagy, respectively. Instead, K6 and K27 ubiquitination seems to have a role in signal transduction rather than substrate degradation [80,82]. Despite its role in chaperone-dependent quality control, STUB1 levels are not increased after proteotoxic stress and overexpression of STUB1 was even shown to be toxic in flies [83], suggesting STUB1 E3 ubiquitin ligase activity is regulated through a different mechanism. Balaji et al [84] recently suggested the STUB1 oligomeric state as a mediator of its activity, as the STUB1 dimer and STUB1 monomer exert different activities that regulate substrate ubiquitination to control proteostasis and longevity, respectively. The dimer-monomer transition is regulated by autoubiquitination and chaperone binding, providing a feedback loop to modulate STUB1 activity in response to proteotoxic stress. Notably, it has been also shown that STUB1 is able to mediate the endocytic-lysosomal degradation of regulatory proteins in a chaperone-independent manner [83]. STUB1 is also involved in stress sensing, signal transduction and damage responses [80,81,85,86]. In cells, STUB1 mainly localizes in the cytoplasm but has also been detected in the nucleus and on the cytosolic surface of mitochondria upon stress induction [87,88]. STUB1 is highly expressed in tissues characterized by high metabolic activity and high protein turnover, such as the cardiac and skeletal muscles or the brain [89,90]. Surprisingly, mice lacking STUB1 exhibit normal embryonic development and turnover of known STUB1 substrates, suggesting a functional redundancy among E3 ubiquitin ligases [91,92]. However, STUB1 deficiency is associated with accelerated aging and reduced life span, hinting toward a critical role for proper STUB1 functioning in controlling longevity [91].

### DNAJs: do they have a role in CASA?

As mentioned, HSPAs fulfil their refolding activity thanks to the cooperation of DNAJs and nucleotide exchange factors. However, it is not yet clear how and which members of the DNAJ family take part in CASA complex activities. *In vitro* experiments indicated that the activity of HSPA is determined by its interaction with a specific DNAJ-BAG combination, suggesting that combinatorial assembly with co-chaperones aids in expanding the functional diversity of HSPAs [44]. Evidence of DNAJs interaction with the CASA members has been reported for DNAJB6 [93,94], although its role in the CASA pathway remains uncharacterized. Noteworthy, mutations in genes encoding some members of the DNAJ family have been described to cause very similar phenotypes associated with BAG3 and HSPB8 mutations [95–100].

## The dynamics of CASA in proteostasis maintenance

CASA-mediated degradation begins with client recognition by the chaperones HSPA and HSPB8. It is not yet clear how these chaperones can bind to a wide array of substrates, but it is widely accepted that chaperones recognize misfolded substrates by exposed hydrophobic patches, which would be buried in their native structures. As mentioned, the formation of the CASA complex is mediated by BAG3, which acts as a scaffold for the interaction with the other CASA members and factors involved in client targeting to degradation [15]. Substrates bound to the CASA complex can undergo ubiquitination by STUB1, which allows their sorting toward the autophagic pathway [15]. Once the substrate is ubiquitinated, the autophagic receptor SQSTM1/p62 (sequestosome 1) interacts with the polyubiquitin chain through its ubiquitin-associated (UBA) domain [15]. The role of other selective autophagy receptors in CASA has not been investigated yet. A recent study described the cooperation between SQSTM1, NBR1 (NBR1 autophagy cargo receptor), and TAX1BP1 (Tax1 binding protein 1) in the selective autophagy of ubiquitinated substrates, with SQSTM1 being the major driver of ubiquitin condensate formation [101]. Given the specific contribution of each receptor, CASA likely relies on the initial recruitment of SQSTM1, supported by NBR1 and TAX1BP1 for promoting condensate formation and initiation of autophagosome formation, respectively. Alternatively, since BAG3 possesses putative LIRs, it has been suggested that BAG3 might facilitate autophagy through direct binding to LC3-decorated autophagosomes, although the functional relevance of these regions has not been established yet and requires further investigation [63]. CASA ends with the engulfment of the substrate-bound CASA complex into autophagosomes and subsequent lysosome fusion and cargo degradation.

In specialized cell types, CASA occurs in defined intracellular regions in cooperation with cytoskeletal adaptors proteins of the SYNPO family. For instance, in neuronal processes, the CASA complex assures the clearance of MAPT (microtubule associated protein tau) [56]. Specifically in the postsynaptic compartment, MAPT degradation by CASA is facilitated by the interaction of BAG3 with SYNPO, which mediates autophagosome and lysosome fusion [56]. Similarly, in muscle cells, CASA promotes the clearance of FLNC at the Z-disk. Here, SYNPO2 links the CASA complex to the proteins and membranes involved in autophagosome formation [57].

CASA substrates may be also routed to specialized sites for further processing, such as aggresomes. The aggresome is a stress-induced, juxta-nuclear inclusion body that colocalizes misfolded proteins, molecular chaperones, and components of the UPS at the microtubule organizing center (MTOC), and requires an intact microtubular network [102]. When the proteasome is blocked, aggresome formation is promoted while autophagosome generation is inhibited. Aggresome formation is an early event of proteasome inhibition and is considered a protective response aimed to compartmentalize substrates to be subsequently degraded by the selective autophagy machinery and other components of the PQC. The autophagy receptor SQSTM1 plays a main role in promoting aggresomes formation or autophagic engulfment of substrates, depending on its phosphorylation status [103,104].

The MTOC represents a specialized site for protein degradation, as supported by the constitutive presence of a fraction of proteasomes, chaperones, autophagosomes and lysosomes [105–107]. Once misfolded proteins are actively compartmentalized into aggresomes, their disposal seems to occur mainly through the autophagy lysosomal pathway (ALP). Efficient aggresomal clearance by selective autophagy is facilitated by its proximity to the endoplasmic reticulum (as a source for autophagosomal membranes), actin filaments (for transport of autophagic and lysosomal vesicles), and by the clustering of lysosomes around the aggresome [107–109].

**Figure 4.**
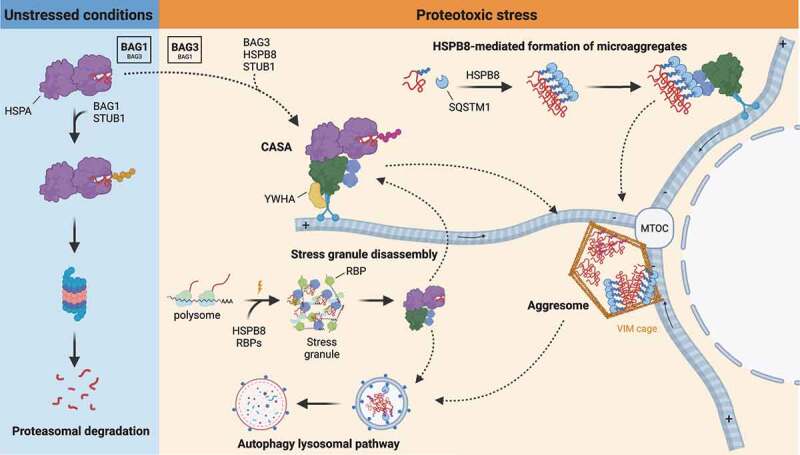
Mechanisms of CASA dynamics and activities. In unstressed conditions, damaged and misfolded substrates are routed to the UPS by HSPA-STUB1-BAG1 complex. Upon proteotoxic stress, HSPA preferentially interacts with STUB1-BAG3 and HSPB8, forming the CASA complex. The CASA complex utilizes the dynein machinery to route substrates to the MTOC, where they are deposited at the aggresome. Alternatively, HSPB8 drives ubiquitinated substrates and SQSTM1 microaggregates formation and routing to the aggresomes. Aggresomes are subsequently processed by the ALP. CASA complex is also involved in granulostasis upon stress, by promoting stress granule disassembly through the extraction of misfolded substrates (and DRiPs) from the stress granule and their routing to ALP degradation.

The CASA complex participates in the routing of substrates to the MTOC. This is achieved by the interaction of the BAG3 PxxP domain with the dynein motor complex and retrograde transport of the whole CASA complex (Figure 4). Alternatively, clients are targeted to aggresomes through HSPB8 direct recognition of SQSTM1-regulated ubiquitinated microaggregates (Figure 4). In this model, HSPB8 activity consists of concentrating misfolded and ubiquitinated substrates into microaggregates and assisting SQSTM1 oligomerization. Next, HSPB8 promotes efficient coupling of BAG3 to SQSTM1 for ubiquitinated microaggregates transport to the MTOC, although the direct physical interaction between HSPB8 and BAG3 seems to be dispensable for aggresome formation. In the proposed mechanism, HSPB8 upstream activity in sequestering ubiquitinated misfolded substrates into spatially defined compartments becomes fundamental under severe proteotoxic stress, when a fast reaction against toxic proteins and activation of adaptive response pathways is required for cell homeostasis [52,110].

As mentioned above, routing of the CASA complex and the bound substrate to the MTOC occurs through a dynein-mediated retrograde active transport along the microtubule network. Indeed, drugs impairing the microtubule network (e.g.: the microtubule-depolymerizing drugs vinblastine or nocodazole) suppress aggresome formation [52,110]. The specific inhibition of retrograde transport, which can be achieved by manipulating the expression of proteins modulating the dynein machinery or by using drugs that inhibit the motor activity of dynein, also blocks the BAG3-mediated routing of the CASA cargo [110–112]. In addition, since BAG3-dynein coupling occurs through the BAG3 PxxP domain, its deletion abrogates dynein interaction and therefore aggresome formation [52]. In contrast, Guilbert et al. [110] observed a rescue of BAG3-mediated aggresome formation upon reintroduction of the BAG3 ΔPxxP mutant in BAG3-depleted cells. This suggests that the contribution of the PxxP domain in aggresome targeting could be compensated for by other mechanisms, such as interactions mediated by the YWHA proteins. As previously mentioned, BAG3-dynein coupling and CASA delivery to the aggresome involve the YWHA proteins, which are engaged by BAG3 through its two RSQS136 and RSQS173 motifs [62]. Of note, BAG3-mediated aggresome formation requires its interaction with HSPB8 and HSPA, as deletion of both IPV motifs or the BAG domain suppresses substrate targeting to the aggresome [52,110]. It must be underlined that, substrate ubiquitination does not seem to be necessary for BAG3-mediated aggresome targeting [52], in line with the observation that many misfolded proteins found in aggresomes are not ubiquitinated [112].

## CASA complex activity and stress signaling pathways

**Figure 5.**
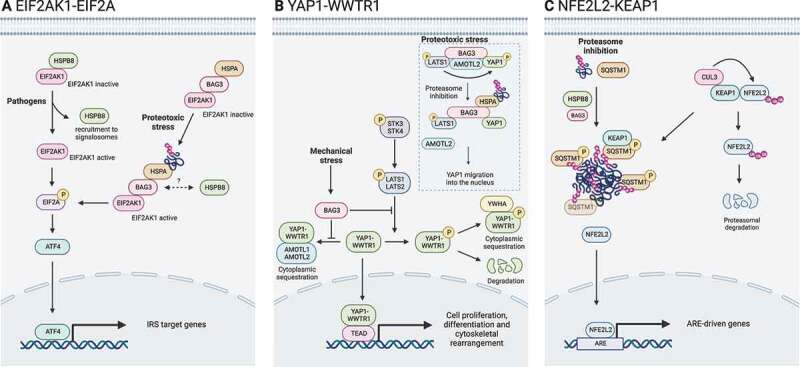
CASA complex in stress signaling pathways. The CASA complex directly regulates signaling pathways involved in the adaptive response to stress. (A) EIF2AK1/HRI-EIF2A signaling axis is activated by pathogens or proteotoxicity. In response to pathogens, HSPB8 is recruited to signalosomes and releases EIF2AK1, which promotes EIF2A phosphorylation. Alternatively, in response to proteotoxic stress, the binding of ubiquitinated substrates to HSPA-BAG3 activates EIF2AK1 signaling. Phosphorylated EIF2A stimulates transcription factors (e.g., ATF4) of the integrated stress response (ISR). (B) Hippo pathway and YAP1-WWTR1 signaling is activated by mechanical stress or proteotoxicity. Upon mechanical stress, BAG3 binds LATS1 or LATS2 and AMOTL1 or AMOTL2, thereby suppressing the cytoplasmic sequestration or degradation of YAP1-WWTR1, allowing their translocation into the nucleus. Alternatively, upon proteotoxic stress, ubiquitinated substrates compete with AMOTL2 for LATS1 binding. AMOLT2 displacement suppresses YAP1 phosphorylation and permits BAG3-mediated YAP1 translocation into the nucleus and expression of its target genes. (C) NFE2L2-KEAP1 signaling axis. In physiological conditions, KEAP1 is bound to NFE2L2 and favors its CUL3-mediated ubiquitination and proteasomal degradation. Upon proteasome inhibition, HSPB8 favors the sequestration of ubiquitinated substrates into SQSTM1 oligomers. Phosphorylated SQSTM1 bodies recruit KEAP1, freeing NFE2L2. Unbound NFE2L2 translocates into the nucleus where it promotes the transcription of stress-responsive genes.

An overload in damaged and misfolded proteins stimulates the expression and the activity of the members of the CASA complex. An increase in damaged and misfolded substrates can be caused by various stress agents, including proteasome impairment, mechanical and oxidative stress. CASA interplays with various signaling pathways that sense harmful events and induce cell adaptive responses. Signaling pathways that are activated in response to stress agents include the integrated stress response (ISR), the Hippo pathway, and the NFE2L21 (NFE2 like bZIP transcription factor 2)-KEAP1 (kelch like ECH associated protein 1) axis (Figure 5).

### EIF2AK1/HRI-EIF2A signaling axis

In response to a variety of stresses, eukaryotic cells activate a common adaptive pathway to adjust their metabolic status, named the integrated stress response (ISR) [113,114]. The core event in this pathway is the phosphorylation of EIF2A/eIF2α (eukaryotic translation initiation factor 2A), which leads to a decrease in general protein synthesis and the induction of a stress-associated transcriptional reprogramming to promote cellular recovery. The phosphorylation of EIF2A is facilitated by a family of five EIF2A kinases: EIF2AK2/PKR (eukaryotic translation initiation factor 2 alpha kinase 2 [activated by double-stranded RNA]), EIF2AK3/PERK (eukaryotic translation initiation factor 2 alpha kinase 3 [responding to endoplasmic reticulum, ER stress]), EIF2AK1/HRI (eukaryotic translation initiation factor 2 alpha kinase 1 [induced by low levels of heme]), EIF2AK4/GCN2 (eukaryotic translation initiation factor 2 alpha kinase 4 [sensing amino acid deficiency]), and MARK2 (microtubule affinity regulating kinase 2 [a serine/threonine kinase implicated in the regulation of microtubule stability that acts as a direct kinase of EIF2A in response to misfolded proteins-induced stress]) in response to various stressors [115]. These kinases respond to a broad range of stresses, including heme deprivation, oxidative and osmotic stress, heat shock and proteasome inhibition, although cytosolic proteotoxicity may represent the overarching trigger for their activation (reviewed in [116]).

The first evidence for the interplay between CASA proteins and the ISR came from the observation that overexpression of HSPB8 and/or BAG3 induced EIF2A phosphorylation, followed by a translational shutdown and the stimulation of autophagy [117]. However, the upstream player(s) converting the overexpression of HSPB8 or BAG3 into a signal responsible for the phosphorylation of EIF2A remained elusive.

During recent years, it has become clear that the CASA complex members form an integral part of the EIF2AK1-EIF2A signaling axis upon proteotoxic stress (Figure 5A). HSPB8 was shown to take part in the cytosolic unfolded protein response (cUPR), an intracellular pathway involved in innate immune signaling [118]. HSPB8 interacts with EIF2AK1 and, upon the formation of large assemblies (signalosomes) in response to pathogens, HSPB8 is recruited to avoid signalosome aggregation and a defective NFKB/NF-κB (nuclear factor kappa B)-mediated immune response. Simultaneously, the dissociation of HSPB8 from EIF2AK1 leads to the phosphorylation of EIF2A and the synthesis of ATF4, which mediates the induction of *de novo* HSPB8 expression to sustain signaling. Notably, the NFKB signaling pathway has also been indicated as an inducer of CASA complex activity and HSPB8 and BAG3 expression [119,120]. The EIF2AK1-EIF2A axis can be activated by aggregating proteins as well, such as SNCA/α-synuclein, suggesting EIF2AK1 could be involved in detecting and restricting the accumulation of cytosolic protein aggregates.

Mukherjee et al. [121] demonstrated the involvement of EIF2AK1 in clearing cytosolic protein aggregates upon proteasome inhibition. Interestingly, silencing of EIF2AK1 resulted in decreased levels of BAG3 and HSPB8, suggesting that EIF2AK1 functions in cytosolic proteostasis through the CASA complex. Based on the key role of HSPB8 in EIF2AK1-dependent control of innate immune receptor scaffolds, they proposed a two-fold relationship between EIF2AK1 and autophagy: (i) the displacement of HSPs from EIF2AK1 to cytosolic protein aggregates may contribute to the activation of EIF2AK1, as previously suggested in the case of the HSPA8 and HSPC [122–126], and (ii) the regulation of the expression of autophagy genes (likely through the EIF2A-ATF4 axis).

Extending the work of Mukherjee et al. [121], Patel et al. [127] investigated the involvement of the HSPA-BAG3 complex, previously shown to regulate responses of stress kinases to proteotoxicity [58], and further unraveled the mechanisms of EIF2AK1 activation by proteotoxicity. In response to the inhibition of the HSPA-BAG3 complex, transcriptomic and subsequent pathway analyses predicted the activation of UPR-related genes triggered by the phosphorylation of EIF2A. Based on their experimental data, they proposed a role for the HSPA-BAG3 complex in transmitting the cytoplasmic proteotoxicity signal to the EIF2AK1-EIF2A axis. In the resting state, EIF2AK1 is kept in an inactive form by the HSPA-BAG3 complex. Upon pharmacological disruption of the HSPA-BAG3 complex or inhibition of the proteasome, EIF2AK1 becomes active and cooperates with BAG3 for the phosphorylation of EIF2A.

### Hippo pathway and YAP1-WWTR1 signaling

The CASA complex also responds to mechanical stress, thus regulating cytoskeletal rearrangements through the Hippo pathway and YAP1 (Yes1 associated transcriptional regulator)-WWTR1/TAZ (WW domain containing transcription regulator 1) signaling. The mammalian Hippo pathway is a kinase cascade in which the STK3 (serine/threonine kinase 3) and STK4 phosphorylate and activate LATS1 (large tumor suppressor kinase 1) and LATS2. In turn, LATS1 and LATS2 phosphorylate YAP1 and WWTR1 proteins, resulting in their retention in the cytoplasm. YAP1 and WWTR1 are transcription co-activators that modulate the expression of genes involved in proliferation and structural maintenance, including FLN (filamin) proteins [57].

Filamins function as mechanosensors and undergo unfolding during cellular straining. This is particularly relevant in muscle cells, where the FLNC (filamin C) isoform is a target for CASA complex-mediated degradation, but also a target gene of the YAP1 and WWTR1 proteins. With these mechanisms, CASA assures FLNC turnover. The role of the CASA complex in promoting cytoskeletal reorganization is also fundamental in non-striated muscle cells, which express the FLNA isoform. An example is represented by the immune cells, in which the cytoskeletal rearrangement is necessary for migration and adhesion of these cell types to the damaged tissues and during inflammation [57].

The YAP1 and WWTR1 proteins are in an inactive state when retained in the cytoplasm. YAP1 and WWTR1 localize in the cytoplasm when bound by the WW domains of the LATS1 and LATS2 and AMOTL1 and AMOTL2 proteins. Upon mechanical stress, BAG3 is bound to LATS1 and LATS2 and AMOTL1 and AMOTL2 through its WW domain, releasing the two co-activators that migrate into the nuclei [57] (Figure 5B).

Notably, when HSPA is pharmacologically inhibited, the CASA complex activity is disrupted and the YAP1 and WWTR1 signaling remains active and normally translocates in the nucleus. Instead, when BAG3 is silenced, YAP1 and WWTR1 nuclear translocation is reduced. In muscle cells, the deregulation of the BAG3 and YAP1 and WWTR1 signaling ultimately results in alterations in myoblasts differentiation and fusion [128].

It has been also reported that BAG3 can directly interact with YAP1 through its N-terminal domain including the WW domain, resulting in the formation of a complex including YAP1, LATS1 and AMOTL2, all taking interaction with BAG3. The deletion of the WW domain completely prevented LATS1 interaction and reduced the association of YAP1 with BAG3 [59]. This complex is responsive to proteotoxic stress. Indeed, the proposed mechanism is based on the increase in ubiquitinated substrates at the CASA complex, which compete with AMOTL2 for the binding to LATS1, as the first possesses a ubiquitin moiety that is recognized by the UBA domain of the latter. AMOTL2 dissociation prevents YAP1 phosphorylation by LATS1. In this case, phosphorylated YAP1 retains interaction with BAG3, which assists YAP1 translocation into the nucleus assisted by the YWHA protein [59].

Another member of the Hippo pathway, the STK38 seems to be involved in the activity of the CASA complex, but in a kinase-independent mechanism. Indeed, it has been shown that STK38 directly interacts with BAG3 causing a loss of interaction with HSPB8 and SYNPO2, which ultimately results in the abrogation of FLN degradation through CASA [129].

### NFE2L2-KEAP1 signaling axis

Another signaling pathway that is related to the CASA complex is the NFE2L2-KEAP1 axis (Figure 5C). KEAP1 is recruited in SQSTM1 oligomers, which are regulated by HSPB8 upon stress. KEAP1 sequestration by SQSTM1 correlates with the release and stabilization of the NFE2L2 transcription factor [110]. Thus, the activated NFE2L2 enhances the expression of genes involved in stress adaptation and survival, by binding to antioxidant response elements. Intriguingly, NFE2L2 activation induces BAG3 expression, as well, together with autophagic receptors (e.g., SQSTM1) [130,131], suggesting a positive feedback mechanism on CASA during the cell stress response.

## CASA crosstalk with the UPS

To maintain a healthy proteome, the pathways of the proteostasis network cooperate at various levels. As mentioned, HSPAs represent a central hub for substrate routing to folding processes or degradative systems. When substrates are routed for degradation through ubiquitination by STUB1, their fate toward autophagy or the UPS is dependent on the interacting co-chaperones. Under physiological conditions, the preferred route involves ubiquitination and proteasome-mediated degradation. However, the narrow core tunnel of the proteasome forms a biophysical limitation for the processing of stable higher-order structures such as oligomers or aggregates, which often cannot be sufficiently unfolded. Moreover, an overload in misfolded substrates can overwhelm the proteasome, necessitating the activation of complementary systems to minimize the accumulation of toxic misfolded protein species. One of these adaptive responses comprises the switch from a high BAG1-dependent proteasomal activity to a BAG3-mediated autophagy system for protein degradation [34,132]. Similarly, when BAG3-mediated autophagy is blocked, the expression of BAG1 is enhanced and substrates are subjected to BAG1-mediated proteasomal degradation [111]. The BAG3:BAG1 ratio thus indicates whether selective autophagy or proteasomal degradation is the preferred route for misfolded protein clearance in a cell. Like BAG3, BAG1 is expressed ubiquitously [133]. Besides the BAG domain, it possesses a ubiquitin-like domain, which permits the routing of polyubiquitinated substrates to the proteasome system. Factors that modulate the BAG1 to BAG3 switch are acute stress or aging. Indeed, aged cells show higher levels of BAG3 and lower levels of BAG1 with respect to young cells [134]. The BAG1 to BAG3 switch and vice versa represents an example of a compensatory activity that reflects the integrated response of the PQC system to overcome proteotoxic stress.

## The role of the CASA complex in extracellular secretion

The CASA complex may utilize alternative pathways for substrates disposal, which are based on CASA complex secretion into the extracellular environment [135]. It has been postulated that CASA complex secretion into extracellular vesicles might represent an alternative attempt of cells to clear proteotoxic and aggregating substrates when the intracellular degradative pathways are impaired [136]. In support of this notion, the toxic fragments of TARDBP/TDP-43 (TAR DNA binding protein) associated with amyotrophic lateral sclerosis (ALS) have been detected in extracellular vesicles containing CASA complex members and their secretion increased upon combined UPS and autophagy blockage [136]. The hypothesized mechanisms through which CASA members may be secreted rely on the internalization into exosomes through an LC3-dependent manner or the secretion into extracellular vesicles by direct budding of the plasma membrane [136,137].

## CASA complex beyond autophagy

### Granulostasis

Stress granules (SGs) are cytoplasmic membrane-less organelles formed by stalled small ribosomal subunits and mRNAs, translation initiation factors and other ribonucleoproteins that assemble through a liquid-liquid phase separation process [138–140]. In cells, SGs formation is a physiological response to stress. Indeed, stress triggers a translational arrest by inducing phosphorylation of the initiator factor EIF2A and polyribosomes disassembly, releasing mRNAs and ribonucleoproteins that are then temporarily sequestered in SGs [141]. With polyribosome disassembly, newly synthesized polypeptides are also released, including aberrant protein products characterized by co-translational misfolding, premature termination events, amino acids misincorporation or other defects [142]. This fraction represents the so-called defective ribosomal products (DRiPs), which are the main source of misfolded proteins in cells. DRiPs are generally promptly recognized by members of the PQC system (e.g., HSPA or VCP [valosin containing protein]) and targeted for degradation [143,144]. However, upon PQC impairment, DRiPs accumulate into SGs, compromising their dynamics and making them persistent [145]. Interestingly, only a small fraction of SGs depends on autophagic clearance, whereas the majority is disassembled in a chaperone-dependent/autophagy-independent way. Members of the CASA complex (HSPB8-BAG3-HSPA) were shown to participate in safeguarding SGs composition and dynamics (referred to as granulostasis) by recognizing and promoting the disassembly of aberrant SGs, preventing them from becoming persistent [146]. The HSPB8-BAG3-HSPA chaperone complex acts in a two-step process: first, HSPB8 dissociates from BAG3-HSPA to be recruited by SGs in response to acute stress (e.g., proteasome inhibition or oxidative stress). Here, HSPB8 exerts its holdase activity, preventing the irreversible aggregation of misfolded substrates or DRiPs inside SGs. Second, upon stress recovery, HSPB8 recruits the BAG3-HSPA machinery, which extracts misfolded substrates from SGs. Hence, misfolded substrates are subjected to degradation or targeted to the aggresome for subsequent disposal [146,147]. In some cells, persistent SGs are transported to the aggresome, and cleared in an autophagy-dependent manner [147]. However, as mentioned before, autophagy did not seem to be the preferred pathway of stress granule clearance, as (i) most SGs disassemble rapidly when the stress subsides and (ii) persistent SGs continuously decreased in size during transport to the aggresome, suggesting they are still being partially disassembled in a chaperone-mediated fashion. This is in line with the study by Jain et al. [148], which proposed that SGs consist of a dynamic shell surrounding a more stable core structure, which might have become resistant to disassembly due to extensive aberrant interactions with misfolded proteins [147]. Whether the HSPB8-BAG3-HSPA/HSP70 complex participates in the final autophagic degradation of aggresome-localized persistent SGs was not addressed, although this is reasonable given the role of CASA in the degradation of aggresomal substrates.

### Cell division

During cell division, cytoskeletal structures undergo complex and massive remodeling. During mitosis, actin structures rearrange forming a stiff actin cortex, which accompanied cell retraction and rounding; thin actin fibers, called retraction fibers, attach to the substrate and guide spindle orientation parallel to the substrate. Mitosis progression and chromosome segregation require the correct orientation of the mitotic spindle. Mitosis ends with cytokinesis: the two daughter cells separate thanks to the actomyosin ring that tightens forming an intracellular bridge between the two cells. To finalize cell division, this intracellular bridge must thin until cleavage and this event occurs with actin depolymerization.

HSPB8 and BAG3 contribute to the homeostasis of actin structures during cell division. BAG3 is hyperphosphorylated at the mitosis entry and is associated with HSPB8, SQSTM1 and HDAC6 (histone deacetylase 6). During mitosis, BAG3 and HSPB8 localize at the separating centrosomes or in the perinuclear material before nuclear envelope breakdown and then at centrosomes at spindle poles during metaphase through anaphase transition. Notably, the depletion of BAG3 decreases the incidence of cell division, slows down the mitotic process, and causes an increase in nuclear abnormalities such as micro- and multinucleation, which are hallmarks of improper mitosis and cytokinesis. In fact, BAG3-depleted cells show an aberrant positioning of the mitotic spindle and misalignment of chromosomes. Defects in mitotic spindle orientation and chromosome segregation are also detectable in HSPB8 and SQSTM1-depleted cells, because of actin cortex disorganization. Noteworthy, mitotic spindle dynamics guided by BAG3 and HSPB8 seem to be independent of the HSPA interaction [149].

During cytokinesis, the depletion of BAG3 and HSPB8 associates with a higher incidence and persistence of abnormally thick and long intracellular bridges, suggesting a role of these proteins in actin dynamics in cytokinetic structures. HSPB8 depletion alone is associated with an aberrant accumulation of F-actin at the intracellular bridges, and this alteration is reverted by limiting the pool of free G-actin or by inhibiting the actin related protein 2/3 (ARP2/3) complex, which promotes actin polymerization. Noteworthy, the ARP2/3 complex component ARPC2 has been found in BAG3 interactome studies [150]. It has been suggested that HSPB8-BAG3 complex activity in cytokinesis involves facilitating the autophagic clearance of actin-based structures. Indeed, the autophagic activator drug rapamycin was able to prevent aberrant F-actin accumulation in HSPB8-depleted cells [151].

## The physiological and pathological roles of the CASA complex

### Homeostasis in skeletal and cardiac muscles and the nervous system

**Figure 6.**
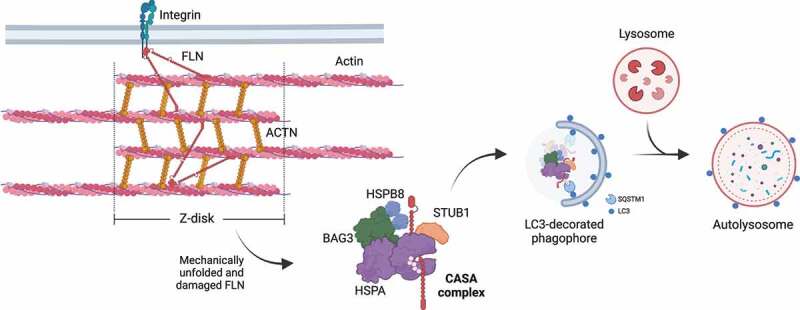
CASA-mediated degradation of Z-disk component FLNC. Mechanically unfolded and damaged forms of FLN are recognized and degraded by the CASA pathway. Here, BAG3 closely cooperates with HSPB8 (the “holdase”) and regulates the ATP-dependent chaperone cycle of HSPA, necessary for substrate processing. STUB1-mediated ubiquitination and subsequent recruitment of the autophagic receptor SQSTM1 initiates autophagosome formation, followed by lysosomal fusion for degradation.

The term CASA was first used to define a BAG3-mediated degradation pathway essential for muscle maintenance [15]. As previously mentioned, BAG3 coordinates the formation of the multicomponent chaperone complex at the Z-disk of skeletal muscle cells, facilitating the degradation of damaged components, such as FLNC (Figure 6) [57]. FLNC is a large dimeric protein that crosslinks actin filaments to membrane proteins [152]. Upon tension, FLNC unfolds, exposing sites for CASA complex recruitment, which promotes its degradation to assure the structural integrity of skeletal muscle cells under physiological conditions [15,57,153].

In the heart, CASA is also involved in the functional and structural maintenance of cardiac muscle cells. In cardiomyocytes, BAG3 and HSPA stabilize the beta subunit of the F-actin capping protein (known as CAPZB/CapZβ1 [capping actin protein of muscle Z-line subunit beta]), regulating the distribution of actin filaments at the Z-disk, while BAG3 depletion associates with myofibrillar disruption upon contraction [154]. Notably, cardiac and skeletal muscle tissue defects have been also reported in various animal models of BAG3 deficiency [16,70,155–157]. HSPB8 deficiency associates with cardiac dysfunction [158].

In the brain, mainly in neuronal cells, CASA exerts a protective function against neurodegeneration. The neuroprotective functions of STUB1 have been investigated in Parkinson disease, Alzheimer disease, ALS and several polyglutamine (polyQ) diseases and are mediated by the ability of STUB1 to form heterocomplexes with both HSPAs and HSPCs and to participate in substrate routing to both the proteasome and autophagy [159–165]. An increase in HSPB8 and BAG3 expression is observed in presence of aggregation-prone proteins, likely to enhance autophagic removal of aggregating substrates [166,167]. Indeed, CASA boosting by HSPB8 or BAG3 overexpression or by pharmacological induction has been proven to decrease the accumulation of the microtubule-associated protein tau in Alzheimer disease, SNCA/α-synuclein in Parkinson disease, the extended polyQ stretch-containing proteins huntingtin and androgen receptor, causative of Huntington and spinal and bulbar muscular atrophy diseases, respectively. Also, CASA-mediated clearance was observed for SOD1 (superoxide dismutase 1) carrying the G93A mutation, the toxic C-terminal fragments of TARDBP, and the dipeptide repeats products that result from the non-canonical repeat-associated non-AUG (RAN) translation of the C9orf72 GGGGCC hexanucleotide expansion, which are linked to ALS-frontotemporal lobar degeneration [69,166,168–174].

### CASA complex members in disease

The relevant role of CASA in muscle and neuronal tissues is emphasized by the identification of mutations in genes encoding CASA members especially in diseases affecting these tissues (Table 1). Notably, no mutations have been reported in cytosolic HSPAs, most likely related to their essential and multifaceted roles in proteostasis. In contrast, several variants and mutations in BAG3 have been associated with diseases, primarily dilated cardiomyopathy, but also myopathy and neuropathy. Although the pathogenicity of several dilated cardiomyopathy variants might be questioned based on the absence of functional studies, a few BAG3 mutations have been deeply investigated in cell and animal models. For instance, the most frequently reported missense mutations affect the proline 209: the p.P209L mutation is causative of autosomal dominant myofibrillar myopathy, cardiomyopathy and severe axonal neuropathy in children [175–178]; the p.P209Q mutation is linked to mild, late-onset myofibrillar myopathy and axonal neuropathy or peripheral neuropathy [179]; the p.P209S mutation is described in axonal Charcot-Marie-Tooth disease [180]. Although these mutations affect the second IPV motif of BAG3, cellular studies indicate that the interaction with HSPB8 is not abolished [181,182]. Instead, the pathogenic mechanisms of BAG3 p.P209 reside in its ability to stall the HSPA machinery in client folding, which results in BAG3 mutant increased aggregation propensity, with relocation of the CASA complex and other PQC factors in aberrant aggresomes, and an alteration of SQSTM1 phosphorylation [110,181,182]. Similar functional alterations are observed in the myofibrillar myopathy-related BAG3 p.P470S, which falls in the BAG domain [181]. Overall, BAG3 p.P209 and p.P470S mutations result in impaired CASA activity and a general alteration of the PQC system, as supported by the presence of abnormal protein accumulation in biopsies from patients carrying one of these two mutations [176,181]. Instead, the dilated cardiomyopathy-related BAG3 p.E455K, which also affects the BAG domain, causes a decrease in the interaction with HSPA and likely affects CASA activity and proteostasis through a different mechanism [183]. Noteworthy, animal models of BAG3 p.P209 mutations recapitulate the human diseases. For instance, aggregation of the human BAG3 p.P209L is observed when overexpressed in zebrafish [155]. Also, a *bag3*^P215L/-^ mouse model (carrying the p.P215L mutation, equivalent to human p.P209L) displays a myopathy phenotype [184].

**Table 1.** Overview of reported disease-causing mutations in BAG3, HSPB8 and STUB1.

Gene	Mutation	Inheritance	Human pathology	Reference
BAG3	R71W	Dominant	DCM	[185]
	R90X	Dominant	DCM	[185]
	I94F	Dominant	DCM	[185,186]
	H109R	Dominant	DCM	[185]
	P115S	Dominant	DCM	[185]
	A121X	Dominant	DCM	[185]
	R123X	Dominant	DCM	[185,187,188]
	P209L	Dominant	MFM, axonal neuropathy, myopathy, CMT	[155,175,176,178,185,189–196,264]
	P209Q	Dominant	MFM, axonal sensorimotor polyneuropathy	[179]
	P209S	Dominant	CMT	[180,197]
	R218W	Dominant	DCM	[198–200]
	R218GfsX89	Dominant	DCM	[185]
	H243TfsX64	Dominant	DCM	[187]
	Q251RfsX56	Dominant	DCM	[185]
	R258W	Dominant	MFM	[175]
	A262T	Dominant	DCM	[185]
	M306X	Dominant	DCM	[201]
	R309X	Dominant	DCM	[183,202]
	Q353RfsX10	Dominant	DCM	[203]
	G379AfsX45	Dominant	DCM	[203]
	P380S	Dominant	DCM	[204]
	S385QfsX56	Dominant	DCM	[183]
	P407L	Dominant	DCM	[183]
	T451X	Dominant	DCM	[203]
	E455K	Dominant	DCM	[183,203]
	L462P	Dominant	DCM	[200]
	V468M	Dominant	DCM	[183]
	P470S	Dominant	MFM with axonal polyneuropathy	[181,205]
	R477H	Dominant	DCM	[185]
HSPB8	P90L	Dominant	dHMN	[234]
	N138T	Dominant	dHMN	[234]
	K141E	Dominant	dHMN, CMT, MFM	[206,207,232,235]
	K141N	Dominant	dHMN, CMT	[206–208,232,234]
	K141M	Dominant	dHMN	[234]
	K141T	Dominant	CMT	[207,233]
	Q170Gfs*45	Dominant	Axial and distal myopathy	[239]
	P173Sfs*43	Dominant	Peripheral motor neuropathy and a rimmed vacuolar myofibrillar myopathy	[235,240]
	T176Wfs*38	Dominant	Rimmed vacuolar myopathy	[242]
	T194Sfs*23	Dominant	Limb-girdle rimmed vacuolar myopathy	[241]
STUB1	E28K/K144X	Recessive	SCAR16	[209]
	G33S	Dominant	SCA48	[210]
	N34S	Digenic	SCA17-DI	[244]
	R35S/I227P*fs11	Recessive	SCAR16	[211]
	F37L	Dominant	SCA48	[212]
	A45V	Dominant	SCA48	[213]
	A46P	Dominant	SCA48	[214]
	Y49C	Dominant/Digenic	SCA48/SCA17-DI	[213,214,244]
	A52G	Dominant	SCA48	[215]
	I53T	Dominant	SCA48	[212]
	P57L	Dominant	SCA48	[214,216]
	N65S	Dominant/Recessive	SCAR16/SCA48	[209,214]
	R66P	Dominant	SCA48	[213]
	A67T	Dominant	SCA48	[216]
	A67V	Digenic	SCA17-DI	[244]
	C69Y	Dominant	SCA48	[214]
	Q74P	Dominant	SCA48	[214]
	A79P	Dominant	SCA48	[214]
	A79T/A79D	Recessive	SCAR16	[217]
	A86T	Digenic	SCA17-DI	[244]
	Q92X	Digenic	SCA17-DI	[244]
	G101R	Dominant	SCA48	[214]
	A113D	Dominant	SCA48	[214,218]
	R119X/I294 F	Recessive	SCAR16	[245]
	A120V/M211T	Recessive	SCAR16	[214]
	L123V	Recessive	SCAR16	[217]
	N130I/W147C	Recessive	SCAR16	[89]
	F131L	Digenic	SCA17-DI	[244]
	D133V	Digenic	SCA17-DI	[244]
	D134del	Digenic	SCA17-DI	[244]
	K143del	Dominant	SCA48	[214,218]
	K145Q	Digenic	SCA17-DI	[244]
	K145Q/Y230Cfs*9	Recessive	SCAR16	[219]
	K145Q/P243L	Recessive	SCAR16	[245]
	K145Q/R241W	Recessive	SCAR16	[220]
	K145Q/C232G	Recessive	SCAR16	[220]
	W147*	Dominant	SCA48	[214]
	W147C	Recessive	SCAR16	[89,221]
	R154C	Dominant	SCAR48	[214,218]
	L165F	Recessive	SCAR16	[89]
	L168F	Dominant/Recessive	SCA48/SCAR16	[214,218]
	R182X	Dominant	SCA48	[214]
	D189A	Recessive	SCAR16	[222]
	C199F+D212G	Dominant	SCA48	[214]
	Y207X/S236T	Recessive	SCAR16	[89]
	M211I/E238X	Recessive	SCAR16	[223]
	M211T	Recessive	SCAR16	[214]
	D212G	Dominant	SCA48	[214]
	S216Ffs*5	Recessive	SCAR16	[224]
	Q217X	Digenic	SCA17-DI	[244]
	R222K	Dominant	SCA48	[214]
	R225X	Dominant/Digenic	SCA48/SCA17-DI	[216,244]
	I227Pfs*11	Recessive	SCAR16	[211]
	P228S	Dominant	SCA48	[210]
	Y230Cfs*9	Dominant/Recessive/Digenic	SCA48/SCA16/SCA17-DI	[216,219,244]
	L231V	Recessive	SCAR16	[214]
	C232G	Dominant/Recessive	SCAR16/SCA48	[213,220]
	C232del	Recessive	SCAR16	[224]
	S236T	Recessive	SCAR16	[89]
	E238X	Recessive	SCAR16	[223]
	M240T	Recessive	SCAR16	[217]
	R241G	Recessive	SCAR16	[224]
	R241W	Dominant/Recessive	SCA48/SCA16	[216,220]
	E242K	Recessive	SCAR16	[225]
	P243L	Dominant/Recessive/Digenic	SCA48/SCAR16/SCA17-DI	[214,244,245]
	C244Yfs*24	Dominant	SCA48	[226]
	T246M	Recessive/Digenic	SCAR16/SCA17-DI	[209,247]
	V264Gfs*4	Dominant	SCA48	[216]
	G265D	Dominant	SCA48	[213,214]
	P274Afs*3	Dominant	SCA48	[216]
	L275Ds*16	Dominant/Digenic	SCA48/SCA17-DI	[216,227,228,244]
	L275Rfs*11	Dominant	SCA48	[214]
	L275V	Recessive	SCAR16	[229]
	L275X	Digenic	SCA17-DI	[244]
	L275P	Digenic	SCA17-DI	[244]
	E278Nfs*9	Dominant/Digenic	SCA48/SCA17-DI	[230,244]
	E278X	Digenic	SCA17-DI	[244]
	N283K	Dominant	SCA48	[214]
	A285D	Dominant	SCA48	[231]
	D288del	Recessive	SCAR16	[213]
	D288X	Digenic	SCA17-DI	[244]
	I294F	Recessive	SCAR16	[245]

Abbreviations: DCM = dilated cardiomyopathy, CMT = Charcot-Marie-Tooth, MFM = myofibrillar myopathy, dHMN = distal hereditary motor neuropathy, SCAR16 = autosomal recessive spinocerebellar ataxia type 16, SCA48 = autosomal dominant spinocerebellar ataxia type 48, SCA17-DI = digenic spinocerebellar ataxia type 17

Like BAG3, HSPB8 mutations have been described in distal hereditary motor neuropathy, axonal Charcot-Marie-Tooth disease, and myopathies. In most of the reported cases, the HSPB8 mutations involve the p.K141 residue (p.K141E/N/T/M) [232–235]. The pathogenic mechanisms associated with HSPB8 p.K141 mutations have been investigated in cells and animal models and consist of: i) mutant HSPB8 aggregation that sequesters other PQC and structural factors; ii) impairment of the autophagic flux; and iii) mitochondrial alterations [232,236]. Indeed, *hspb8*^K141N/K141N^ knockin mice display progressive axonal degeneration and muscle atrophy, with degenerating organelles and accumulating mitochondria, decreased autophagy and HSPB8 aggregation [237]. *Drosophila* models overexpressing the HSPB8 ortholog Hsp67Bc carrying the p.R126E or p.R126N mutations (corresponding to human HSPB8 p.K141E or p.K141N) show normal muscle performance, but display histopathological hallmarks of muscle dysfunction, with myofibrillar disorganization, mitochondrial defects, alterations at the neuromuscular junctions, and protein aggregation [238]. Instead, myopathies are the predominant pathological conditions of a group of HSPB8 frameshift mutations [239–242]. In this case, HSPB8 haploinsufficiency has been suggested to negatively affect the CASA pathway [239,240]. However, since the *hspb8* KO mouse model does not display signs of muscle alterations, other pathogenic mechanisms rather than the sole HSPB8 deficiency are likely involved in the development of the disease [237].

Differently from BAG3 and HSPB8, mutations in *STUB1* have been linked to spinocerebellar ataxias, diseases characterized by degeneration of the cerebellum and cognitive impairment. Spinocerebellar ataxias related to *STUB1* mutations display a recessive (SCAR16) or dominant (SCA48) inheritance pattern and are variable in terms of onset, progression, and symptoms [243]. In addition, heterozygous mutations in *STUB1* have been also described in a digenic form of SCA17 (SCA17-DI), in patients also carrying intermediate polyQ expansions in TBP (TATA-box binding protein) [244]. In some cases, SCA associated with STUB1 mutations has been considered one pathological feature of a multisystemic disease, in which ataxia may be accompanied by hypogonadism (as in Gordon Holmes syndrome), pyramidal tract damage, dementia and hyperkinetic movement disorders [245]. Mutations occur in the whole structure of the STUB1 protein, affecting differently the secondary structure, stability, dimerization, oligomerization, or ubiquitination activity. In some cases, mutations in STUB1 result in a complete loss of ubiquitination activity and aggregation-prone behavior [246]. However, most STUB1 mutations or variants lack a deep characterization, which might also be difficult to assess for the digenic forms of SCA. Thus, as for several BAG3 variants reported, studies should be addressed to define the pathogenicity of STUB1 variants. Notably, the *stub1* KO mouse model is viable, but displays loss of Purkinje cells in the cerebellum and manifests ataxia, cognitive impairment, and hypogonadism [247]. This evidence suggests that STUB1 activities cannot be fulfilled by other E3 ubiquitin ligases in some neuronal cell types and, possibly, in gonadotropic cells, although it is not clear whether the hypogonadism is due to hypothalamic or pituitary defects [247].

## The CASA complex as a pharmacological target

Pharmacological targeting of the CASA complex has been under investigation for the treatment of various pathological conditions. Notably, drugs that upregulate or downregulate the expression of CASA members or CASA activity may result beneficial in some diseases, while detrimental in others. This is the case for cancer, in which CASA members may exert dual and opposite roles in disease progression, as reviewed in [248–252].

Instead, several neurodegenerative and neuromuscular diseases may benefit from therapeutic agents that potentiate CASA complex activity, based on its involvement in targeting misfolded and aggregating proteins to autophagic disposal. To enhance the CASA activity, the upregulation and stabilization of the CASA members represent the current strategy. However, it should be considered that HSPAs and STUB1 also act in other pathways of the PQC besides CASA. Thus, pharmacological targeting of HSPAs and STUB1 specifically in the CASA complex is unlikely feasible. Nevertheless, an enhancement of HSPAs (and STUB1) might result in a synergistic potentiation of the different proteostasis pathways in which HSPAs are involved, and molecules activating these chaperones are under clinical evaluation in the treatment of neurodegenerative diseases [253,254]. Conversely, targeting HSPB8 and BAG3, which are the limiting factors of the CASA complex, represents a specific approach to potentiate the CASA pathway.

As mentioned, proteostasis impairment (e.g., pharmacological inhibition of the proteasome) enhances HSPB8 and BAG3 expression [46,255–257]. Similarly, heat shock, infections, and heavy metals cause an upregulation of the CASA complex members, to restore cell homeostasis [48,258,259]. While the therapeutic potential of stressors in neurodegeneration and muscle disease is certainly questionable, the study of these toxic insults allowed the identification of transcription factors that are involved in HSPB8 and BAG3 regulation, such as NFKB and HSF1 [47,50,119,120,259].

Compounds that have been described to upregulate the transcription of HSPB8, but not BAG3, with established outcomes in neurodegenerative disease are colchicine and doxorubicin, which have been proven to induce also other autophagy genes and the clearance of protein aggregates related with ALS in cell models [260]. Although the mechanism of action of colchicine in HSPB8 upregulation is still undefined, its efficacy is currently under investigation in a clinical trial on ALS patients [261]. Similarly, it is not yet clear how another autophagic inducer can stimulate the expression of HSPB8 and BAG3. This is the case for trehalose, which potentiates autophagy through the activation of TFEB (transcription factor EB) in response to transient lysosomal permeabilization [262]. Interestingly, while trehalose, colchicine and doxorubicin modulate TFEB activation or expression, the upregulation of HSPB8 and/or BAG3 observed in response to these drugs seems not to be mediated by TFEB itself [260,262].

It is clear that the upregulation of HSPB8 and BAG3 can offer a promising therapeutic option in neurodegeneration. However, in the case of diseases caused by mutations in CASA complex members, the molecular basis of CASA dysfunction must be carefully considered. For instance, the observations of BAG3 coaggregation with the CASA complex members and other PQC factors in disease models of BAG3 mutations suggest that upregulation of CASA may be detrimental to disease progression [181,182]. In this case, an attempt to improve the BAG3 mutant phenotype has been made by targeting the protein-protein interaction between BAG3 and HSPAs using the HSPAs inhibitors JG98 or YM01 [181]. However, while this approach was successful in cell models and patient fibroblast, the use of JG98 resulted to be toxic to cardiac and skeletal muscle cells, which are the cell types affected by BAG3 mutations [263]. Alternatively, to avoid the collateral effects due to unspecific pharmacological targeting of HSPAs, a strategy based on the potentiation of the PQC system can be envisaged to favor CASA member aggregates removal. Indeed, autophagic induction by metformin resulted in BAG3 aggregates clearance and improvement of the myopathic phenotype in a BAG3 p.P209L zebrafish model [264]. Since aggregation is a common feature of HSPB8 mutations as well [265], the enhancement of autophagy could represent a valuable strategy to get rid of both the substrates bound to the CASA complex and the dysfunctional CASA complex itself, avoiding a worsening of the cell homeostasis as a consequence of sequestration of main factors of the PQC system.

## Conclusions and future perspectives

In past decades, considerable progress has been made in understanding how cells mitigate proteotoxic stress. The CASA complex targets misfolded proteins to degradation, either directly by interacting with the autophagic receptor SQSTM1, or by temporally sequestering misfolded proteins in aggresomes for subsequent degradation. The maintenance of functional CASA activity (and PQC in general) is relevant in cellular aging processes. Aging associates with an overall decline of proteostasis and an increasing burden in damaged protein products, a considerable issue especially for those tissues characterized by a low or absent renewal potential (e.g., post-mitotic cells like neurons or muscle cells). Notably, STUB1 expression increases with cellular senescence, while its depletion associates with premature aging in mice. Also, with aging, an increased BAG3:BAG1 ratio suggests a preferential routing of damaged proteins toward autophagy, likely to compensate for the inefficient capacity of the UPS in processing the increasing amounts of misfolded substrates. Pharmacological targeting of the CASA complex may therefore be considered as a promising therapeutic approach in age-related diseases, further substantiated by multiple studies showing that CASA boosting could counteract protein aggregation in both cellular and animal models of neurodegenerative diseases. However, the upregulation of CASA members may exert deleterious effects in other pathological conditions. For instance, in some types of cancer, CASA members displayed a pro-tumoral activity. In addition, it has been shown that some mutations in CASA members are causative of diseases because of a toxic gain of function of the mutated protein. These observations suggest that the underlying mechanisms through which the CASA complex acts in diseases should be deeply dissected and that caution should be used in promoting pharmacological therapies that boost the CASA complex in age-related conditions. Taken together, the protective role of CASA in the maintenance of neuronal and muscle tissues strongly supports the notion that the CASA complex itself represents a promising candidate for drug discovery in neuromuscular and neurodegenerative diseases. Nevertheless, growing evidence suggests that the CASA complex plays multiple and independent roles besides selective autophagy, which makes this pathway relevant in cell homeostasis.
